# Factors affecting the yield of bio-oil from the pyrolysis of coconut shell

**DOI:** 10.1186/s40064-016-1974-2

**Published:** 2016-03-15

**Authors:** Yun Gao, Yi Yang, Zhanbin Qin, Yi Sun

**Affiliations:** Department of Chemical Engineering, North China University of Science and Technology, Tangshan, 063009 Hebei People’s Republic of China

**Keywords:** Coconut shell, Pyrolysis, Coconut oil, Oil yield, Pyrolysis oil

## Abstract

Coconut is a high-quality agricultural product of the Asia–Pacific region. In this paper, coconut shell which mainly composed of cellulose, hemicellulose, lignin was used as a raw material for coconut shell oil from coconut shell pyrolysis. The influence of the pyrolysis temperature, heating rate and particle size on coconut oil yield was investigated, and the effect of heating rate on coconut oil components was discussed. Experimental results show that the maximum oil yield of 75.74 wt% (including water) were obtained under the conditions that the final pyrolysis temperature 575 °C, heating rate 20 °C/min, coconut shell diameter about 5 mm. Thermal gravimetric analysis was used and it can be seen that coconut shell pyrolysis process can be divided into three stages: water loss, pyrolysis and pyrocondensation. The main components of coconut-shell oil are water (about 50 wt%), aromatic, phenolic, acid, ketone and ether containing compounds.

## Background

Energy shortages and environmental contamination are growing problems in modern society. Biomass oil is a natural fuel product which can be obtained from cellulose and hemicelluloses synthesized during photosynthesis (Li and Ying 2009). In this process, CO_2_ and H_2_O are combined to form a green energy alternative to diminishing fossil fuel reserves (Department of Energy 2011; Davis et al. 2009). These materials also have the potential to alleviate worsening environmental pollution. Biomass oil is the fourth largest energy source in the world (Jiang 2002). Biomass oil can be industrially produced on a large scale and will play a major role in China’s future energy development. Optimization of biomass production is needed for the advancement of this energy producing chemical industry (Anastas and Kirchhoff 2002; Kitajima and Yamamoto 2002).

Biomass pyrolysis is a widely used technology for biomass energy production (Patun et al. 2005). Pyrolysis is one of the most economical and promising technologies to convert biomass to liquid fuel products. Pyrolysis will produce a high yield of liquid products when performed under hypoxic conditions, high temperatures (400–500 °C), a short residence time for the volatile component (less than 5 s) and a heating rate of 10–200 °C/min (Balat et al. 2009; Bridgwater 2012; Zhang et al. 2007).

Biomass pyrolysis produces three main products, flammable gas, liquid pyrolysis oil, and solid charcoal. The gas product contains methane, which is widely present in natural gas, biogas, and coal mine gases. It is a high quality gas fuel and an important raw material for the manufacture of syngas and many chemical products. Liquid products can be refined to produce liquid fuels or value-added products, and can also be used as a chemical raw material. The products of liquid biomass pyrolysis manufacturing include adhesives, phenol, and liquid fuel or resin (Trinh et al. 2013). Solid charcoal can be used directly or to produce activated carbon or battery and electrode components.

Biomass pyrolysis technology has been well studied in China (Li et al. 2013). Du et al. (2009) evaluated the pyrolysis of solid wood and pine bark at different heating rates and with different particle sizes to show that increases in the heating rate were associated with thermal gravimetric analysis (TGA) curves and that weight loss rate curves shifted to a higher temperature zone. Tunce and Gerce (2004) studied the effects of heating rate, pyrolysis temperature and gas atmosphere on bio-oil production. The highest oil yield was achieved with a pyrolysis temperature of 550 °C, and heating rate of 7 °C/min. Liu et al. (2012) analyzed the pyrolysis of raw coconut shells using TGA. The pyrolysis of coconut shells was divided into water loss, pyrolysis and pyrocondensation stages.

Coconut is a high-quality agricultural product of the Asia–Pacific region. The major coconut producing area in China is Hainan. Despite the presence of abundant coconut resources in Hainan, coconut processing operations remain under-developed. The pyrolysis of coconut shells yields coconut shell oil, from which value-added products and chemical raw materials can be produced. Coconut shell has a tough natural structure and low ash content, making it ideal for easy physical processing and low environmental impact. Hasanah et al. (2012) analyzed the chemical composition and physical properties of the light and heavy tar resulted from coconut shell pyrolysis. We used TGA to study the effects of pyrolysis temperature, heating rate and particle size on coconut shell oil production. The main components of the coconut oil product were analyzed using Fourier transform infrared spectroscopy (FTIR).

## Methods

Hainan coconut drupe was used as the raw material. The coconut was opened, the eatable portion removed, and the coconut shell collected. The coconut shell was ground and fragments sized using graded sieves. Coconut fragments 0.1–1, 4–8, and 10–20 mm were placed in a digital thermostatic drying oven at 120 °C for 12 h before evaluation.

**Fig. 1:**
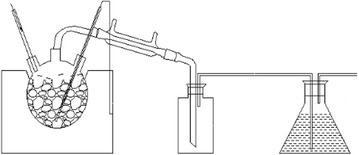
Schematic experimental unit for pyrolysis

100 g of sized coconut shell fragments were placed in a quartz flask. Pyrolysis reactions were carried out in an argon atmosphere using an electric heating mantle. Thermocouple was put in the middle of the coconut shell fragments and connected with the electric heating mantle to control the pyrolysis temperature. A heat exchanger condenser consisting of a low-temperature thermostatic bath was used to collect volatile vapor phase products in a collecting bottle. The schematic experimental unit for pyrolysis is shown in Fig. 1.

The coconut shell charcoal was weighed after pyrolysis completed. Coconut oil yield was calculated as:

$${\text{H}} = \left( {{\text{M}}_{1} - {\text{ M}}_{2} } \right) /\left( {{\text{M}}_{3} - {\text{ M}}_{4} } \right) \, \times \, 100\;\%$$

where H is the oil yield in wt%, M_1_ is the mass of the collecting bottle after the experiment in g, M_2_ is the mass of the collecting bottle before the experiment in g, M_3_ is the mass of the raw coconut shell before the experiment in g, and M_4_ is the mass of the raw coconut shell after the experiment in g.

TGA was performed using an integrated thermal analyzer STA449C (NETZSCH, Germany) with 99.99 % nitrogen carrier gas at a rate of 30 mL/min. The samples were heated from room temperature at certain rate to a selected temperature. FTIR analysis of coconut shell oil was performed using an FTIR spectrometer AVATAR 360 (Nicolet, USA).

KF-1A type semi-automatic moisture analyzer was used to analyze the water content in coconut oil product. The Karl Fischer titration was applied in analyzing.

## Results and discussions

### Evaluation of final material temperature on yield

**Fig. 2:**
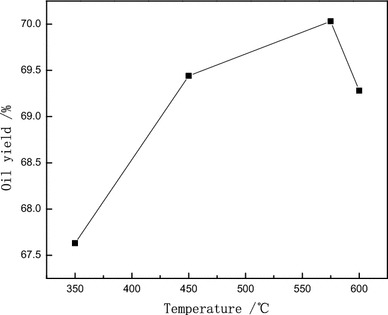
Effect of final pyrolysis temperature on oil yield

A heating rate of 10 °C/min was used to achieve final pyrolysis temperatures of 350, 450, 575, or 600 °C (Fig. 2). The most coconut-shell oil was collected using a final pyrolysis temperature of 450 °C. Higher temperatures were associated with a reduction in the yield of coconut oil. This reduction is thought to be due to the decrease in condensable volatile products found at higher temperatures. Carbonization and the production of non-condensable small-molecular-weight gases are thought to mainly occur at these higher temperatures. Breakdown of coconut oil to small molecular weight non-condensable gas hydrocarbons or hydrogen will reduce the yield of coconut oil. The ideal temperature for coconut oil production will be high enough to drive out the oils, but not high enough to allow secondary degradation of the vaporized oil.

### Evaluation of heating rate

**Fig. 3:**
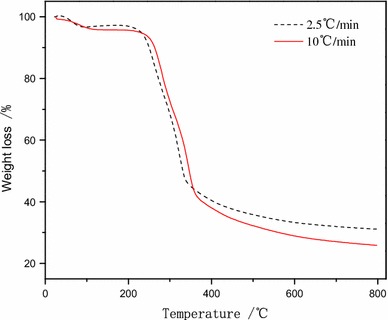
TG curves for raw coconut shell materials at different heating rates

**Fig. 4:**
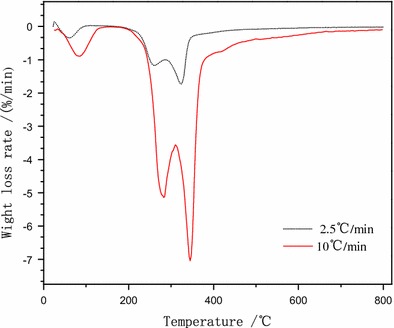
Weight loss rate for raw coconut shell materials using different heating rates

Coconut shell pyrolysis reactions using different heating rates, 2.5 and 10 °C/min to 800 °C, were evaluated. Thermogravimetry (TG) curves and corresponding derivative thermogravimetric (DTG) curves were shown in Figs. 3 and 4.

The TG curves produced with these two heating rates were similar, suggesting the pyrolysis mechanisms were similar (Fig. 3). The TG curves demonstrated three stages of pyrolysis, water loss, pyrolysis and pyrocondensation.

The first stage occurred at 25–200 °C. There were small changes in mass during this stage. Weight loss was mainly due to water loss and structural rearrangements with the release of small molecular weight compounds. Water loss in this stage consisted of unbound and bound water.

The second stage occurred at 200–600 °C. The pyrolysis of coconut shell to yield non-condensable small-molecular-weight gases and condensable large molecular weight volatile components resulted in significant changes in the sample mass. The main components of coconut shell are hemicellulose, cellulose, and lignin. Pyrolysis reactions break down these three compounds into lower molecular weight components. Hemicellulose is the most volatile of the three compounds and lignin has a benzene ring, making it the most difficult and last to decompose. The largest weight loss occurred in this stage, mostly between 220 and 450 °C.

The third stage occurred at 600–800 °C. The main reaction here was pyrocondensation and the beginning of carbonization. The C–H and C–O bonds in the residual organic coconut shell material were cleaved to generate non-condensable volatile components. The mass change in this stage was extremely small.

The DTG curves from the two heating rates showed three peaks (Fig. 4). A small peak was seen from room temperature to 150 °C. This area corresponded to water loss from the raw material. The two subsequent peaks were due to the different pyrolysis characteristics of hemicellulose, cellulose, and lignin. Hemicellulose is the most unstable of these compounds. The second peak was formed due to the pyrolysis of hemicellulose, with a maximum weight loss rate from 250 to 300 °C. The third peak was seen with heating from 320 to 350 °C and was mainly due to the pyrolysis of cellulose. Lignin is most resistant to decomposition, with a small pyrolysis rate over a wide temperature range. Very little weight loss was seen from 500 to 600 °C, suggesting little pyrolysis occurred. The weight loss curve was flat above 575 °C, suggesting no further pyrolysis occurred. No more coconut shell oil was produced in this range.

The more rapid heating rate was associated with a slight shift of the TG curve to a higher temperature. The higher heating rate resulted in a more vigorous pyrolysis reaction. The higher heating rate was associated with greater weight loss in the coconut shell and more complete thermal cracking of the contained oils (Fig. 4).

**Fig. 5:**
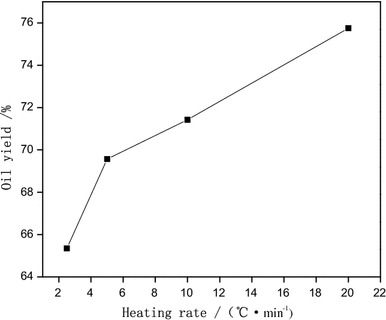
Effect of heating rate on oil yield

The coconut oil yield of different particle sizes was evaluated at heating rates of 2.5, 5, 10, and 20 °C/min to achieve a final pyrolysis temperature of 575 °C (Fig. 5). Increasing the heating rate of any particle size was associated with increased coconut oil yield.

### Evaluation of particle size

Coconut shell fragments less than 1, 4–8, and 10–20 mm were evaluated using a heating rate of 10 °C/min to achieve a final pyrolysis temperature of 575 °C (Table 1). The highest yield was seen with intermediate sized particles. The change in yield with size was thought to be related to different heat transfer rates in the different sized materials. The smallest particles had rapid heat transfer, a high number of secondary cracking reactions with conversion of oil to non-condensable volatile gases, and decreased yield of oil. Large particles took the longest to heat and the presence of the carrier gas prevented these particles from achieving the desired pyrolysis temperature. This incomplete heating was associated with lower yields. Intermediate sized particles had the best heat transfer and least number of secondary cracking reactions, leading to the best yield.

**Table 1:** Particle size of raw materials versus oil yield

Particle size (mm)	<1	4–8	10–20
Oil yield (wt%)	66.98	71.42	61.14

### Coconut oil composition

**Fig. 6:**
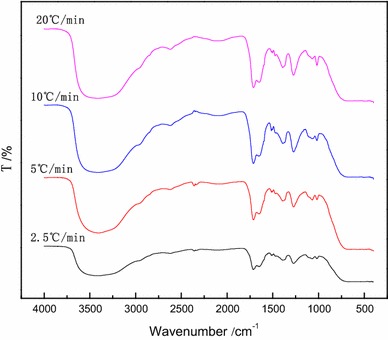
IR spectra of coconut-shell oil at different heating rates

Infrared (IR) spectra in the spectral range of 400–4000 cm^−1^ were obtained. O–H stretching vibrations occur from 3650 to 3200 cm^−1^. The strong absorption peak seen from 3500 to 3200 cm^−1^ was attributed to the presence of O–H groups (Fig. 6). The wide and strong absorption peak at around 3400 cm^−1^ was attributed to intermolecular hydrogen bonding shifting the O–H stretching vibrations to lower wavelengths. The O–H groups in organic acids have increased hydrogen bonding and their absorption peaks are shifted to lower wavelengths, usually around 2600 cm^−1^ (seen in Fig. 6). The absorption peak at 2960 cm^−1^ was attributed to asymmetric stretching vibrations of –CH_3_ groups and the absorption peak near 1390 cm^−1^ was attributed to symmetrical bending motions of –CH_3_. The absorption peak at 1715 cm^−1^ was due to the presence of –C=O groups. This finding suggests that coconut oil contains aromatic ketones. The absorption peak at wavelength 1640 cm^−1^ was assigned to C=C stretching. The absorption peaks at 1515 and 1460 cm^−1^ were attributed to C=C stretching vibrations in the benzene ring. The absorption peak at about 1270 cm^−1^ was attributed to asymmetric stretching vibrations of the aromatic ether bonds = C–O–C. The absorption peaks at around 1065 and 1020 cm^−1^ were attributed to symmetrical stretching vibrations of aromatic ethers. Together, these findings suggest the main components of coconut-shell oil are aromatic, phenolic, acid, ketone and ether containing compounds.

It was shown from Fig. 6 that absorption intensity of –OH increased with the increasing of heating rate, which showed that the amount of phenolic increased in coconut-shell oil. C=O (aromatic) increased with the increasing of heating rate, so aromatic substances in coconut-shell oil was also influenced by heating rate.

### Water content in coconut oil

Water contents of coconut oil in different process conditions was shown in Table 2. It can be seen from the results that large amount of water (about 50 wt%) was existed in the coconut oil products which was different under different process conditions. Water was generated because chemical reactions between volatiles during pyrolysis which was mainly effected by heating rate and final pyrolysis temperature. As the final pyrolysis temperature increased, the water content in coconut oil increased.

**Table 2:** Water content of coconut oil in different process conditions

Heating rate/°C/min	Final temperature/°C	Water content/wt%
2.5	575	58.23
5	575	46.65
10	575	50.29
20	575	51.19
10	350	40.31
10	450	45.26

## Conclusions

In summary, we have demonstrated that the pyrolysis temperature, heating rate and particle size were the main factors affecting the yield of bio-oil from the pyrolysis of coconut shell. The maximum oil yield of 75.74 wt% (including water) were obtained at final temperature of 575 °C, heating rate of 20 °C/min and particle size of 5 mm for the coconut-shell. The main components of coconut-shell oil are water (about 50 wt%), aromatic, phenolic, acid, ketone and ether containing compounds.
